# 17β-Estradiol Does Not Designate Non-Sex-Specific Early Ventricular Arrhythmia in Acute Myocardial Infarction, in Contrast to C-Reactive Protein

**DOI:** 10.3390/ijms27020970

**Published:** 2026-01-19

**Authors:** Niya E. Semedzhieva, Adelina Tsakova, Vesela Lozanova, Petar I. Atanasov, Dobrinka Dineva

**Affiliations:** 1 Clinic of Internal Medicine, University Emergency Medicine Hospital ‘Pirogov’, 1606 Sofia, Bulgaria; 2 Clinical Laboratory Department, University Hospital ‘Sofiamed’, 1680 Sofia, Bulgaria; 3 Department of Biochemistry, Medical University, 1431 Sofia, Bulgaria; vlozanova@medfac.mu-sofia.bg; 4 Clinical Laboratory Department, University Emergency Medicine Hospital ‘Pirogov’, 1606 Sofia, Bulgaria

**Keywords:** acute myocardial infarction, heart rate, QTcmax, QTcd, 17β-estradiol, E2/T

## Abstract

Despite the evidence from experimental studies that endogenous hormones have sex-related effects on action potential duration, the relationship between gonadal steroids and ventricular repolarization in acute myocardial infarction (AMI) is not clear. We tested the hypothesis that endogenous 17β-estradiol (E2) and 17β-estradiol-to-testosterone ratio (E2/T) are associated with inflammation, influencing the occurrence of early ventricular arrhythmia (VA) in AMI. Electrocardiographic (ECG) repolarization indices, including resting heart rate (HR), corrected QT (QTc) interval, QTc minimum (QTcmin), QTc maximum (QTcmax), and QTc dispersion (QTcd), along with E2, total T, and the ratio of E2 to T (E2/T), were measured and analyzed after percutaneous coronary intervention in 86 patients (36 women, 41.9%). In a non-specific sex analysis, the incidence of early VA in the course of AMI was determined by the ejection fraction of the left ventricle (OR 0.876, *p* = 0.054), and by the peak levels of plasma C-reactive protein (OR 1.026, *p* = 0.077). Endogenous plasma 17β-estradiol tended to be higher in cases with early ventricular arrhythmia (124.5 ± 79 vs. 181 ± 192.8, *p* = 0.089). 17β-estradiol levels were significantly predicted by C-reactive protein (OR 1.050, *p* = 0.042). This study found that reduced systolic function of the left ventricle and higher peak CRP levels are associated with endogenous plasma 17β-estradiol in the acute phase of MI, and predicted the risk of early in-hospital ventricular arrhythmia.

## 1. Introduction

Comparing the electrocardiographic characteristics of middle-aged healthy subjects reveals sex-based differences in cardiovascular electrophysiology. In general, women have faster resting heart rates and longer rate-corrected QT intervals, which are measures of myocardial repolarization on surface electrocardiograms [1,2,3].

Prolongation of the corrected QTc interval occurs as a result of acute ischemia [4]. In population-based studies and among patients with coronary disease, a prolonged QTc interval is associated with an increased risk of adverse cardiovascular events and all-cause mortality [5], with some studies suggesting that the strength of this association is influenced by sex [5,6]. QT interval dispersion (QTd) is a non-invasive method of measuring regional inhomogeneities in ventricular refractoriness [7], with higher values of dispersion related to increased incidence of ventricular arrhythmias, nonfatal myocardial infarction, and noncardiac death [8]. QTd has also been demonstrated to increase during myocardial ischemia and decrease with the reversal of the ischemic state following angioplasty [5,9]. In the chronic phase of myocardial infarction, QT dispersion is significantly increased in patients with the highest risk for sudden cardiac death [4] and future ischemic heart attacks [10]. In addition, healthy men have greater QTd values and are more likely to experience ventricular arrhythmias than women [11].

The progression of heart failure also follows sex-specific models [12], and abnormalities in ventricular repolarization are valuable in sex-specific prediction of acute episodes of heart failure [12]. The pathology of acute coronary disease is influenced by biological sex, with sex-specific models demonstrating better discriminative power for predicting outcomes after AMI [13]. In addition, studies on patients with coronary artery disease have shown that women have lower rates of inducible sustained ventricular arrhythmia (VA) than men and are less likely to experience ventricular arrhythmia than men in cases of ischemic cardiomyopathy [14,15,16].

Studies showed that the hearts of female individuals of different mammalian species showed significantly lower IKr and IKs densities compared to those of males [17], with cardiomyocytes in female humans expressing lower levels of various genes responsible for cardiac repolarization [17]. In addition, hormonal differences between males and females have been shown to directly influence the QT interval and risk of TdP [17,18,19]. High estradiol concentrations prolong the action potential duration (APD) in a concentration-dependent manner by altering ICaL, IKr, IK, Ito, and IK1 ionic currents, while testosterone within the physiological range rapidly decreases the APD, primarily by enhancing IKs and suppressing ICaL [18]. The maximum QTc interval and the risk of particular arrhythmias are decreased in pregnancy, with hormonal status being one probable explanation [20]. In addition, the progesterone reduction and estrogen increase that occur in the postpartum period may contribute to arrhythmogenic effects in vulnerable LQTS patients [21]. Contraceptive medications (first- and second-generation oral contraceptives) produce hormonal changes that predispose users to shorter QTc intervals compared to non-users (high progesterone-to-estrogen ratios) [22]. Moreover, modifying the levels of endogenous sex hormones during therapy for certain cancers has also been shown to affect the QT interval duration [23,24]. Female sex is known to be an independent risk factor for the development of TdP in individuals with congenital long QT syndrome and acquired long QT syndrome (LQTS). However, the lower beta-adrenergic receptor density and reduced activity of protein kinase A signal transduction in the hearts of females have been found to limit the positive inotropic effect under beta-adrenergic stimulation, which may explain why women are less prone to severe arrhythmias [18].

The alternating levels of sex hormones during inflammatory disease correlate with an increase in aromatase activity in adipose tissue. Acute myocardial infarction (AMI) is characterized by a significant surge in the levels of inflammatory molecules and oxidative stress compounds in the systemic circulation [25,26], and acute cardiomyocyte necrosis in the infarcted heart activates cell signaling systems, triggering an intense inflammatory response [26]. Current knowledge about the relationship of gonadal steroids with ventricular repolarization and the risk of tachycardia in AMI is limited, despite widely known sex-related differences in repolarization in healthy individuals. Our hypothesis is that early ventricular arrhythmia in AMI is associated with 17β-estradiol levels regardless of biologic sex and is associated with systemic inflammatory molecules and mediators.

### Objectives

The objective of this study was to analyze the association between E2 and the E2/T ratio with inflammatory molecules, indices of ventricular repolarization, and the rate of early ventricular arrhythmia in patients with acute myocardial infarction.

## 2. Results

In total, 11 cases of arrhythmia were detected (12.8%), with a VT incidence of 8.6% (n = 7). Recurrences of continuous VT necessitating specific anti-arrhythmic therapy with amiodarone were only found in three cases. The characteristics of the patients are presented in Table 1.

**Figure 1:**
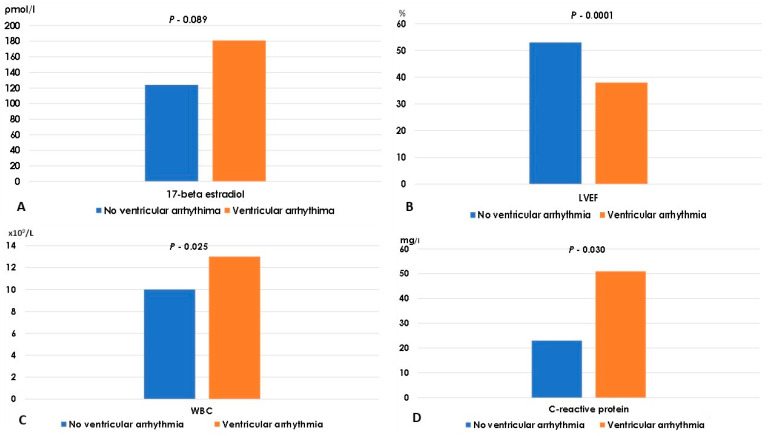
Difference in 17β-estradiol (panel (**A**)), left ventricular ejection fraction (panel (**B**)), white blood cell count (panel (**C**)) and C-reactive protein (panel (**D**)) in patients without ventricular arrhythmia versus patients with ventricular arrhythmia. Legend: LVEF, left ventricular ejection fraction; WBC, white blood cell count.

**Figure 2:**
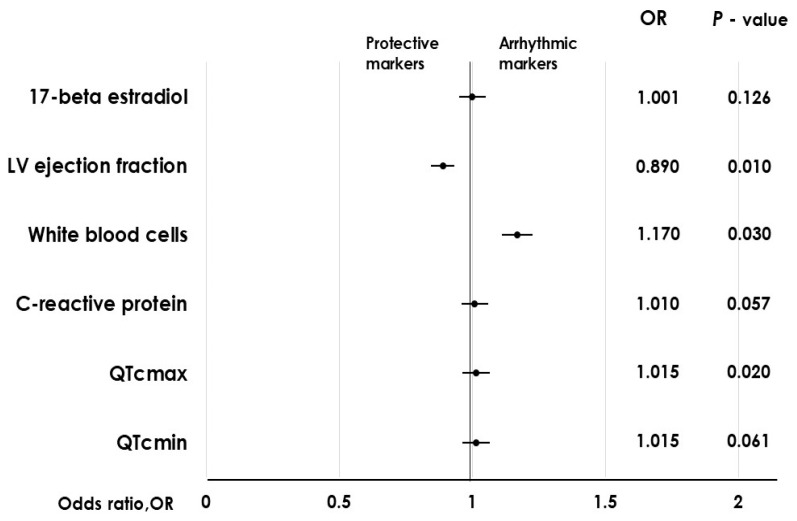
Early ventricular arrhythmia and its covariates. Legend: LV, left ventricular ejection fraction; QTcmax, maximum QTc; QTcmin, minimum QTc.

The incidence of VA in the early course of AMI was found to be correlated with the ejection fraction of the left ventricle and the peak plasma concentrations of CRP, which serves as an inflammatory marker (Table 2; Figure 1 and Figure 2). Endogenous plasma 17β-estradiol exhibited an association with the incidence of early ventricular arrhythmia (Table 2).

EF was inversely associated with 17β-estradiol, the Gensini score, peak WBC, peak CRP, and peak cardiac enzymes, but was only correlated with the severity of CAD (in general) after adjusting for significant covariates (Table 3).

CRP was positively related to cardiac enzymes and was predicted to lead to peak CK-MB levels in the multivariate analysis (Table 4).

The highest peak plasma C-reactive protein concentrations were an independent marker of the highest 17β-estradiol levels in the acute phase of MI (Table 5).

Neither E2 nor inflammation, LVEF and CAD extent predicted the longest maximal repolarization duration in the non-sex-specific analysis of the presented group of patients with AMI (Table 6).

The levels of E2 and E/T were significantly different only for the group with stable coronary disease (AMI vs. stable CAD: age, 66.8 ± 12.2 vs. 68.9 ± 13.5 years, *p* = 0.541; E2: 130.6 ± 96.1 vs. 99.3 ± 33.4 pmol/L, *p* = 0.017; E2/T: 0.15 ± 0.51 vs. 0.04 ± 0.04, *p* = 0.048). In general, the control individuals were younger and had insignificantly lower E2 compared to the patients with AMI (AMI vs. controls: age, 66.8 ± 12.2 vs. 60.1 ± 12.3 years, *p* = 0.086; E2: 130.6 ± 96.1 vs. 112.4 ± 102.1 pmol/L; E2/T: 0.15 ± 0.51 vs. 0.18 ± 0.45).

## 3. Discussion

In the non-sex-specific analysis, the incidence of early VA in the course of AMI exhibited a correlation with the systolic function of the left ventricle and the peak levels of plasma C-reactive protein. In general, endogenous plasma 17β-estradiol was higher in the cases with early ventricular arrhythmia. 17β-estradiol levels in the acute phase of MI were associated with (but not an independent indicator of) the lowest EFs. 17β-estradiol levels were predicted by the peak plasma concentrations of the C-reactive protein.

In our study, the longer initial maximal duration of repolarization, a correlate of ventricular arrhythmia in AMI, is predicted neither by E2 nor by other characteristics of acute CAD (Gensini score, peaks in white blood cell count and in hsTnT).

Previous studies have shown that in patients with documented ventricular arrhythmia, women are more likely to have nonischemic cardiomyopathy, and men are more likely to have coronary disease and ischemic cardiomyopathy [14]. These sex-specific characteristics of cardiovascular pathology can be explained by the earlier development of coronary disease in men than in women [27]; this is associated with the antioxidative effect of estradiol up to menopause, which is unique to women [28]. Our results suggest that there is a relationship between estradiol, inflammatory markers, and the incidence of ventricular arrhythmia in the context of acute coronary disease. However, this is a small observational study, so the results are only hypothesis-generating.

A few recent studies on VA demonstrate a link between aging, estradiol receptor modulation, and an altered myocardial state after cessation of gonadal hormone secretion. A recent study on VA concluded that during experimental AMI, the increased rate of ischemia-driven early VA in AMI was caused by decreased myocardial estrogen receptor antioxidant activity. Fulvestrant, an ER downregulator, was effective in reducing early ventricular arrhythmia in an age- and sex-specific manner [29]. The antiarrhythmic effect of fulvestrant has been proven, with studies showing that it inhibits hyperactivated L-type calcium channels during heart failure [29,30]. Oxidative stress contributes to cardiac arrhythmia through at least two mechanisms: by increasing functional heterogeneities secondary to changed cardiac myocyte metabolic activity (1) and via abnormal conduction induced by inflammatory pathways (2) [31]. A possible reason for this issue is that estradiol substitution after ovariectomy changes myocardial lipid metabolism in a way that significant increases triglyceride content in the myocardium, despite no increase in body weight [32]. Our results do not suggest that oxLDL is associated with repolarization indices. However, these results are statistically underpowered due to the relatively small number of patients whose oxLDL levels were measured and the impossibility of conducting a sex-specific sub analysis.

Many basic studies have demonstrated that inflammatory cytokines (TNF-alpha, IL-1, and IL-6) induced by reactive oxygen species lead to changes in the expression and function of potassium and calcium channels, predisposing patients to a prolonged action potential duration and re-entry ventricular arrhythmias [33,34]. Circulating inflammatory cytokine levels are correlated with QTc duration in a variety of acute and chronic conditions, including AMI [25,26], rheumatoid arthritis [35,36], COVID-19 infection, and neoplastic diseases [37,38,39,40]. In our study, although E2 levels were increased in parallel with the intensity of the inflammatory response to the infarcted myocardium, E2 was not associated with prolonged ventricular repolarization. Thus, E2 appears to be a surrogate marker of the severity of AMI and systemic inflammation, rather than an independent arrhythmogenic factor, in absence of statistically significant association.

A small number of studies suggest that the contribution of E2 to the occurrence of ventricular arrhythmia is complex and genetically determined. Several mutations in the potassium channel, which lead to altered stoichiometry of its subunits, alter IK currents and are associated with LQTS [41]. E2 inhibits the same complex of subunits of the human potassium channel (Kv7.1/KCNE1), further altering IK currents, and some LQTS-associated mutations lead to substantial loss of potassium channel function upon E2 exposure. In contrast, Kv7.1/KCNE1’s reduced or lack of response to E2 in other LQTS-associated mutations suggests that E2 may not be an additional QT-prolonging factor [41].

### Limitations 

The limitations of this study are the small cohort size and the small number of cases with ventricular arrhythmia, which diminishes the statistical power of the analysis and precludes analyses on additional variables related to ventricular arrhythmia (e.g., medications, PCI). Several conclusions rely on *p*-values in the 0.05–0.10 range in a small cohort. Thus, there is high risk of type I error. In the view of only 11 patients who have experienced VA, our multivariable models should be interpreted as exploratory only, not as confirmatory. Another limitation is that the changes over time in hormone and inflammatory markers were not evaluated.

## 4. Materials and Methods

The resting heart rate (HR), corrected QT (QTc) interval, QTc minimum (QTcmin), QTc maximum (QTcmax), QTc dispersion (QTcd), and plasma concentrations of endogenous sex steroids (total 17β-estradiol (E2, Elecsys Estradiol II ECLIA kit, Roche Diagnostics GmbH, Mannheim, Germany), total testosterone (T, Elecsys Testosterone II ECLIA kit, Roche Diagnostics GmbH, Mannheim, Germany) and the plasma levels of oxidized low-density lipoproteins (oxLDLs, OxiSelect Human Oxidized LDL ELISA Kit, Cell Biolabs, Inc., San Diego, CA, USA) and inflammatory markers (white blood cell count [WBC] and C-reactive protein [CRP], CRPHS, Cardiac C-Reactive Protein (Latex) High Sensitive, Roche Diagnostics GmbH, Mannheim, Germany) were measured in 86 (44.5% women) patients after percutaneous coronary intervention (PCI, 88.3%, n = 76) or conservative therapy. The patients were admitted to University Hospital ‘Alexandrovska’ with a diagnosis of AMI from 2011 to 2014, and electrocardiographic (ECG) markers were assessed at hospital admission prior to PCI or conservative therapy.

Cases of ventricular arrhythmia (VA) were defined as recurrent continuous or non-continuous ventricular tachycardia, or ventricular premature complexes (Lown class III). Cases were detected during conventional electrocardiographic monitoring using PCI and during patients’ stay in the intensive care unit. No patients were monitored using cardiac monitor placement or a Holter ECG monitoring system.

The exclusion criteria included a diagnosis of bundle-branch block, left ventricular hypertrophy, paced rhythm, and/or a QTc interval that cannot be measured by all ECG leads. Patients diagnosed with secondary hypogonadism or diseases of the adrenal and pituitary glands were also excluded. Additional exclusion criteria included diagnosis with acute infectious diseases, chronic inflammatory disease, known or suspected neoplastic processes, surgical procedures, and trauma experienced within two weeks before hospital admission. Participants were required to refrain from using hormone or immune therapies for six months prior to and during the study. All women in the study are with postmenopausal status. The hormone and inflammatory marker sampling have been carried out within 6 h of PCI or in the cases of nonobstructive CAD within the first 48 h of the hospital stay.

A total of 15 patients in whom coronary disease was excluded by coronary angiography and 15 patients with stable ischemic heart disease served as control patients.

This study complies with the Declaration of Helsinki and was approved by the ethics committee of Medical University, Sofia. All patients and controls provided written informed consent to participate in the study. The study was registered in the UK’s clinical study registry, ISRCT, registration number ISRCTN62480360.

### 4.1. ECG Analysis

The heart rate and average QTc interval were assessed using automatic records (ECG system; Schiller AT-100), and QTcmin and QTcmax were measured manually by one investigator.

Standard 12-lead ECGs were recorded at a paper speed of 25 mm/s and achieved a gain of 10 mm/mV. The QT interval was measured via the standardized technique [42] and corrected with Bazett’s formula [4]. The corrected QT dispersion was calculated as the difference between the maximum and minimum corrected QT intervals [42].

The mean absolute difference between the first and second measurements of one investigator was 1.0 ± 41.3 ms, with a correlation coefficient of 0.605 (*p* = 0.005) for QTcmin, 4.0 ± 31.5 ms (*p* = 0.003, r = 0.627) for QTcmax, and 2.0 ± 37.2 ms (*p* = 0.107; r = 0.371) for QTc dispersion.

### 4.2. Immunologic Methods

Venous blood samples were drawn after 12 h of fasting, added to EDTA sample tubes, centrifuged at 5000 rpm for 20 min, and stored at −20 °C until analysis. An electrochemiluminescent immunoassay (ECLIA) was used to measure sex hormones in plasma [43]. An enzyme-linked immune assay (ELISA) was used for quantitation of the plasma concentrations of oxLDL [44].

### 4.3. Statistical Analysis

The trial data were managed using SPSS software for Windows version 19.0 and MedCalc statistical software version 23.2.0 (MedCalc Software Ltd., Ostend, Belgium). The results are expressed as the means ± SDs and percentages, and statistical significance was defined as a difference of less than 0.05. The statistical analysis included a χ^2^ test, Fischer’s exact test, paired- and unpaired-sample *t* tests, a Mann–Whitney U test, and correlation and regression analyses.

## 5. Conclusions

This study found that reduced systolic function of the left ventricle and higher peak CRP levels are both correlates of endogenous plasma 17β-estradiol in the acute phase of MI, and predicted the risk of early in-hospital ventricular arrhythmia.

## Figures and Tables

**Table 1:** Demographic profile, comorbidities, and laboratory test results of patients.

Variable	
Ventricular arrhythmia, n (%)	11 (12.8)
Sex (men/women), n (%)	50 (58.1)/36 (41.9)
Age, years	71.8 ± 8.3
Hypertension, n (%)	86 (100)
DM, n (%)	31 (36)
Dyslipidemia, n (%)	69 (80.2)
HR, bpm	76.7 ± 16.9
QTc, msec	439.3 ± 35.3
QTcmin, msec	386.8 ± 44.6
QTcmax, msec	484.2 ± 60.8
QTcd, msec	97.4 ± 45.1
E2, pmol/L	129.9 ± 99.1
E2/T	0.07 ± 0.12
EF, %	51.8 ± 11.6
NSTEMI, n (%)	39 (45.4)
STEMI, n %	46 (53.5)
Gensini score	42.5 ± 35.7
WBC, ×10^9^/L	10.5 ± 4
CRP mg/L	23.4 ± 35.3
oxLDL, mg/dL	8.7 ± 6.0
CK, U/L	889.1 ± 1232.6
CK—MB, U/L	96.1 ± 141.4
hsTnT, ng/mL	2.4 ± 3.3
Percutaneous coronary intervention, n (%)	76 (88.3)
Rhythm- or rate-control therapy at presentation	
β-blocker, n (%)	34 (39.5)
Amiodarone, n (%)	3 (3.4)

Legend: DM, diabetes mellitus; HR, heart rate; QTc, corrected heart rate repolarization period; QTcmin, minimum QTc; QTcmax, maximum QTc; QTcd, dispersion of repolarization; E2, endogenous 17β-estradiol; E2/T, estradiol-to-testosterone ratio; EF, ejection fraction; STEMI, ST elevation myocardial infarction; WBC, white blood cell count; CRP, C-reactive protein; oxLDL, oxidized low-density lipoprotein; CK, creatine kinase; CK-MB—MB fraction of creatine kinase; hsTnT—high-sensitive troponin T.

**Table 2:** Association of heart rate and repolarization indices with endogenous 17β-estradiol, inflammatory markers, and oxLDL, and with VA incidence.

Variable	No VA	VA	*p*-Value	Odds Ratio	Univariate	Regression	Odds Ratio	Multivariate	Regression
					95%CI	*p*-Value		95%CI	*p*-Value
Male patients	43 (86)	7 (14)	NS						
Female patients	32 (88.9)	4 (11.1)	NS						
Age, years	65.9 ± 12.8	64.9 ± 8.2	0.806	0.999	0.950–1.040	0.760			
STEMI	39 (84.8)	7 (15.2)	NS						
HR	76.7 ± 16.1	77.4 ± 24.9	0.955	1.003	0.960–1.047	0.910			
QTcmin	383.7 ± 41	417.7 ± 67.9	0.053 *	1.015	0.999–1.032	0.061 *	1.018	0.992–1.075	0.172
QTcmax	478.8 ± 55.3	538.7 ± 88.8	0.012 **	1.015	1.002–1.028	0.020 **	1.006	0.988–1.024	0.532
QTcd	95.1 ± 41	120.1 ± 75.8	0.162	1.010	0.996–1.025	0.173			
E2	124.5 ± 79	181 ± 192.8	0.089 *	1.000	1.000–1.010	0.129			
E2/T	0.08 ± 0.13	0.05 ± 0.06	0.513	0.016	0.000–309.2	0.412			
EF	53.6 ± 10.8	38.8 ± 9.6	0.0001 **	0.890	0.820–0.950	0.001 **	0.876	0.704–1.003	0.054 *
Gensini score	41.2 ± 35.8	47.6 ± 33.3	0.591	1.010	1.000–1.030	0.139			
WBC	10.1 ± 3.8	12.9 ± 5.1	0.025 **	1.170	1.010–1.350	0.033 **	0.823	0.539–1.003	0.364
CRP	22.7 ± 35.9	50.5 ± 48.6	0.031 **	1.010	1.000–1.030	0.057 *	1.026	0.997–1.056	0.077 *
oxLDL	8.8 ± 6.3	8.4 ± 4.3	0.839	0.999	0.870–1.120	0.837			

Legend: STEMI, ST elevation myocardial infarction; HR, heart rate; QTcmin, minimum QTc; QTcmax, maximum QTc; QTcd, dispersion of repolarization; E2, endogenous 17β-estradiol; E2/T, estradiol-to-testosterone ratio; EF, ejection fraction; WBC, white blood cell count; CRP, C-reactive protein; oxLDL, oxidized low-density lipoprotein. ** and * denote significant associations and those with a trend.

**Table 3:** Variables indicating reduced LV ejection fraction.

Ejection Fraction	Q4	Q1	*p*-Value	Odds Ratio	Univariate	Regression	Odds Ratio	Multivariate	Regression
n-38/13					95% CI	*p*-Value		95% CI	*p*-Value
Age	65.7 ± 13.3	69.6 ± 10.8	0.506	1.000	0.990–1.040	0.353			
E2	109.4 ± 72.3	168.1 ± 184.4	0.013 **	1.004	0.998–1.010	0.131			
E2/T	0.08 ± 0.13	0.11 ± 0.13	0.117	5.700	0.090–368.7	0.414			
CK	407.7 ± 1081.8	1081.8 ± 1270.8	0.005 **	1.001	1.000–1.002	0.018 **	1.000	1.000–1000	0.647
CK-MB	49.7 ± 51.1	126.3 ± 137.9	0.046 **	1.010	1.002–1.018	0.018 **	1.010	0.980–1.040	0.592
hsTnT	1.2 ± 2.3	3.9 ± 4.5	0.033 **	1.290	1.050–1.590	0.016 **	1.170	0.860–1.600	0.305
Gensini score	28.7 ± 24.8	65.8 ± 41.6	0.013 **	1.030	1.010–1.060	0.003 **	1.030	1.000–1.060	0.065 *
WBC	9.6 ± 3.3	12 ± 4.4	0.031 **	1.180	1.010–1.390	0.043 **	0.970	0.750–1.240	0.841
CRP	13.1 ± 13.7	33.7 ± 51.3	0.022 **	1.030	1.010–1.060	0.060 *	1.020	0.980–1.060	0.424
oxLDL	9.3 ± 6.6	9.2 ± 4.2	0.953	1.028	0.997–1.059	0.952			

Legend: E2, endogenous 17β-estradiol; E2/T, estradiol-to-testosterone ratio; CK, creatine kinase; CK-MB—MB fraction of creatine kinase; hsTnT—high-sensitive troponin T; WBC, white blood cell count; CRP, C-reactive protein; oxLDL, oxidized low-density lipoprotein. ** and * denote significant associations and those with a trend.

**Table 4:** Variables indicating extreme values of CRP.

CRP	Q1	Q4	*p*-Value	Odds Ratio	Univariate	Regression	Odds Ratio	Multivariate	Regression
n-21/21					95% CI	*p*-Value		95% CI	*p*-Value
Age	67.8 ± 12.6	67.7 ± 10.5	0.979	0.999	0.947–1.054	0.978			
CK	195.4 ± 178.3	195.4 ± 178.3	0.0001 **	1.004	1.001–1.0077	0.007 **	0.999	0.996–1.003	0. 723
CK-MB	49.7 ± 51.1	126.3 ± 137.9	0.046 **	1.010	1.002–1.018	0.018 **	1.098	1.001–1.191	0.026 **
hsTnT	0.6 ± 2.1	3.6 ± 3.9	0.002 **	1.739	1.106–2.736	0.017 **	0.643	0.349–1.185	0.157
Gensini score	36.7 ± 29.7	53.8 ± 36.8	0.101	1.016	0.997–1.036	0.108			
WBC	9.5 ± 3.9	11.4 ± 4.2	0.089 *	1.129	0.960–1.528	0.144			
oxLDL	8.3 ± 4.7	9.7 ± 5.5	0.139	1.062	0.925–1.220	0.395			

Legend: CK, creatine kinase; CK-MB—MB fraction of creatine kinase; hsTnT—high-sensitive troponin T; WBC, white blood cell count; oxLDL, oxidized low-density lipoprotein. ** and * denote significant associations and those with a trend.

**Table 5:** Relationships between variables for 17β-estradiol.

E2	Q1	Q4	*p*-Value	Odds Ratio	Univariate	Regression	Odds Ratio	Multivariate	Regression
n-21/20					95% CI	*p*-Value		95% CI	*p*-Value
Age, years	67.7 ± 11.1	67.9 ± 159.3	0.392	1.004	0.993–1.014	0.495			
E2/T	0.08 ± 0.08	0.09 ± 0.19	0.892	1.500	0.020–113.9	0.855			
CK	394.4 ± 472.1	1116 ± 1257.9	0.021 **	1.001	1.001–1.002	0.047 **	1.010	0.998–1.004	0.587
CK-MB	54.5 ± 69.9	134.7 ± 145.4	0.028 **	1.007	0.999–1.015	0.065 *	1.000	0.979–1.027	0.993
hsTnT	1.3 ± 2.4	2.9 ± 2.8	0.051 *	1.280	0.968–1.693	0.083 *	1.115	0.823–1.510	0.484
Gensini score	35.9 ± 29.2	54.6 ± 29.2	0.092 *	1.017	0.996–1.037	0.114			
CRP	10.5 ± 13.2	49.9 ± 57.2	0.005 **	1.050	1.005–1.098	0.029 **	1.050	1.000–1.100	0.042 **
WBC	8.9 ± 2.3	11.4 ± 4.3	0.027 **	1.248	0.998–1.558	0.052 *	1.250	0.910–1.700	0.160
oxLDL	8.6 ± 6.7	9.5 ± 6.4	0.652	1.020	0.921–1.230	0.700			

Legend: E2/T, estradiol-to-testosterone ratio; CK, creatine kinase; CK-MB—MB fraction of creatine kinase; hsTnT—high-sensitive troponin T; WBC, white blood cell count; CRP, C-reactive protein; oxLDL, oxidized low-density lipoprotein. ** and * denote significant associations and those with a trend.

**Table 6:** Relationships between variables for QTc max.

QTcmax	Q1	Q4	*p*-Value	Odds Ratio	Univariate	Regression	Odds Ratio	Multivariate	Regression
n-11/11					95% CI	*p*-Value		95% CI	*p*-Value
Age	64.5 ± 9.2	62.6 ± 11.8	0.677	0.980	0.900–1.070	0.660			
E2	136.5 ± 85.7	156.3 ± 190.8	0.758	1.000	0.990–1.010	0.745			
E2/T	0.11 ± 0.17	0.05 ± 0.05	0.336	0.010	0.0001–101.7	0.345			
CK	446.4 ± 681.1	1400.6 ± 1527.0	0.209	1.000	0.999–1.001	0.212			
CK-MB	81.6 ± 86.3	146.2 ± 155.7	0.243	1.003	0.996–1.516	0.246			
hsTnT	1.5 ± 1.6	0.6 ± 4.5	0.050 *	1.190	0.934–1.516	0.076 *	1.330	0.860–2.600	0.207
Gensini score	22.8 ± 16.7	63.4 ± 45.7	0.022 **	1.040	1.000–1.090	0.046 **	1.010	0.970–1.060	0.549
EF	54.3 ± 11.9	43.6 ± 13.8	0.068 *	0.940	0.870–1.010	0.079 *	0.950	0.860–1.040	0.269
WBC	8.6 ± 1.6	11.5 ± 2.5	0.004 **	1.970	1.100–3.530	0.023 **	1.560	0.800–3.020	0.192
CRP	12.6 ± 14.8	32.3 ± 45.8	0.199	1.020	0.980–1.060	0.241			
oxLDL	7.5 ± 4	10.2 ± 9.8	0.462	1.060	0.910–1.250	0.459			

Legend: E2, endogenous 17β-estradiol; E2/T, estradiol-to-testosterone ratio; EF, ejection fraction; WBC, white blood cell count; CRP, C-reactive protein; oxLDL, oxidized low-density lipoprotein. ** and * denote significant associations and those with a trend.

## Data Availability

The original contributions presented in this study are included in the article. Further inquiries can be directed to the corresponding author.

## Abbreviations

The following abbreviations are used in this manuscript:

AMI	acute myocardial infarction
STEMI	acute myocardial infarction with persistent ST elevation
NSTEMI	acute myocardial infarction with non-persistent ST elevation
CAD	coronary artery disease
PCI	percutaneous coronary intervention
HR	heart rate
QTcmin	minimal corrected repolarization period
QTcmax	maximal corrected repolarization period
QTcd	dispersion of coronary repolarization
CK	creatine kinase
CPK-MB	muscle–brain fraction of CK
hs TnT	high-sensitivity troponin T
E2	total 17β-estradiol
E2/T	total 17β-estradiol-to-total testosterone ratio
CRP	C-reactive protein
oxLDL	oxidized low-density lipoproteins
HDL	high-density lipoprotein
LDL	low-density lipoprotein
TG	triglycerides
oxLDL	oxidized low-density lipoprotein
WBC	white blood cell
EF	ejection fraction
